# CRISPR/Cas9 Mutagenesis and Expression of Dominant Mutant Transgenes as Functional Genomic Approaches in Parasitic Nematodes

**DOI:** 10.3389/fgene.2019.00656

**Published:** 2019-07-16

**Authors:** James B. Lok

**Affiliations:** Department of Pathobiology, School of Veterinary Medicine, University of Pennsylvania, Philadelphia, PA, United States

**Keywords:** transgenesis, parasitic nematode, CRISPR/Cas9, dominant transgene, mutagenesis

## Abstract

DNA transformation of parasitic nematodes enables novel approaches to validating predictions from genomic and transcriptomic studies of these important pathogens. Notably, proof of principle for CRISPR/Cas9 mutagenesis has been achieved in *Strongyloides* spp., allowing identification of molecules essential to the functions of sensory neurons that mediate behaviors comprising host finding, invasion, and location of predilection sites by parasitic nematodes. Likewise, CRISPR/Cas9 knockout of the developmental regulatory transcription factor *Ss-daf-16* has validated its function in regulating morphogenesis of infective third-stage larvae in *Strongyloides stercoralis*. While encouraging, these studies underscore challenges that remain in achieving straightforward validation of essential intervention targets in parasitic nematodes. Chief among these is the likelihood that knockout of multifunctional regulators like *Ss*-DAF-16 or its downstream mediator, the nuclear receptor *Ss*-DAF-12, will produce phenotypes so complex as to defy interpretation and will render affected worms incapable of infecting their hosts, thus preventing establishment of stable mutant lines. Approaches to overcoming these impediments could involve refinements to current CRISPR/Cas9 methods in *Strongyloides* including regulatable Cas9 expression from integrated transgenes and CRISPR/Cas9 editing to ablate specific functional motifs in regulatory molecules without complete knockout. Another approach would express transgenes encoding regulatory molecules of interest with mutations designed to similarly ablate or degrade specific functional motifs such as the ligand binding domain of *Ss*-DAF-12 while preserving core functions such as DNA binding. Such mutant transgenes would be expected to exert a dominant interfering effect on their endogenous counterparts. Published reports validate the utility of such dominant-negative approaches in *Strongyloides*.

## Introduction

The advent of transgenesis in parasitic nematodes has opened new means of ascertaining the functions of specific genes in these important pathogens by both gain- and loss-of-function approaches. The first successes in this area involved translating methods for transgenesis in *Caenorhabditis elegans* to parasites in the genera *Strongyloides* and *Parastrongyloides*, which were apt subjects given their capacity to undertake one or more generations of free-living development. Transgenesis in *Strongyloides* spp. has enabled targeted mutagenesis by CRISPR/Cas9, and this method has already found application in studies that assigned function to genes regulating morphogenesis and host-finding behavior in infective larvae. Transgenes designed to exert dominant interfering effects over specific genes have also revealed essential functions of these genes in growth and developmental of pre-parasitic larvae of *Strongyloides stercoralis*. Robust new methods for integrative transgenesis in *Brugia malayi* promise to open these and other avenues of functional genomic study in the filariae and potentially in other obligately parasitic nematodes. None of these functional genomic approaches are new to the basic fields of cellular and molecular biology, but their application to parasitic helminths marks a recent innovation that ranks among the salient advances in parasitology in the past two decades. In view of this, the following review discusses these current findings in detail and proposes new directions in their application to increasingly informative and practical studies of gene function in a group of pathogens that impose the risk of morbidity and mortality on a large segment of the world’s population and exact a heavy toll on the health and welfare of domestic animals.

## Transformation with DNA or RNA Constructs is a Prerequisite to Deploying Contemporary Methods for Assessment of Gene Function in Parasitic Nematodes

### Approaches to Transgenesis in Clade III and IV Parasitic Nematodes

**Figure 1:**
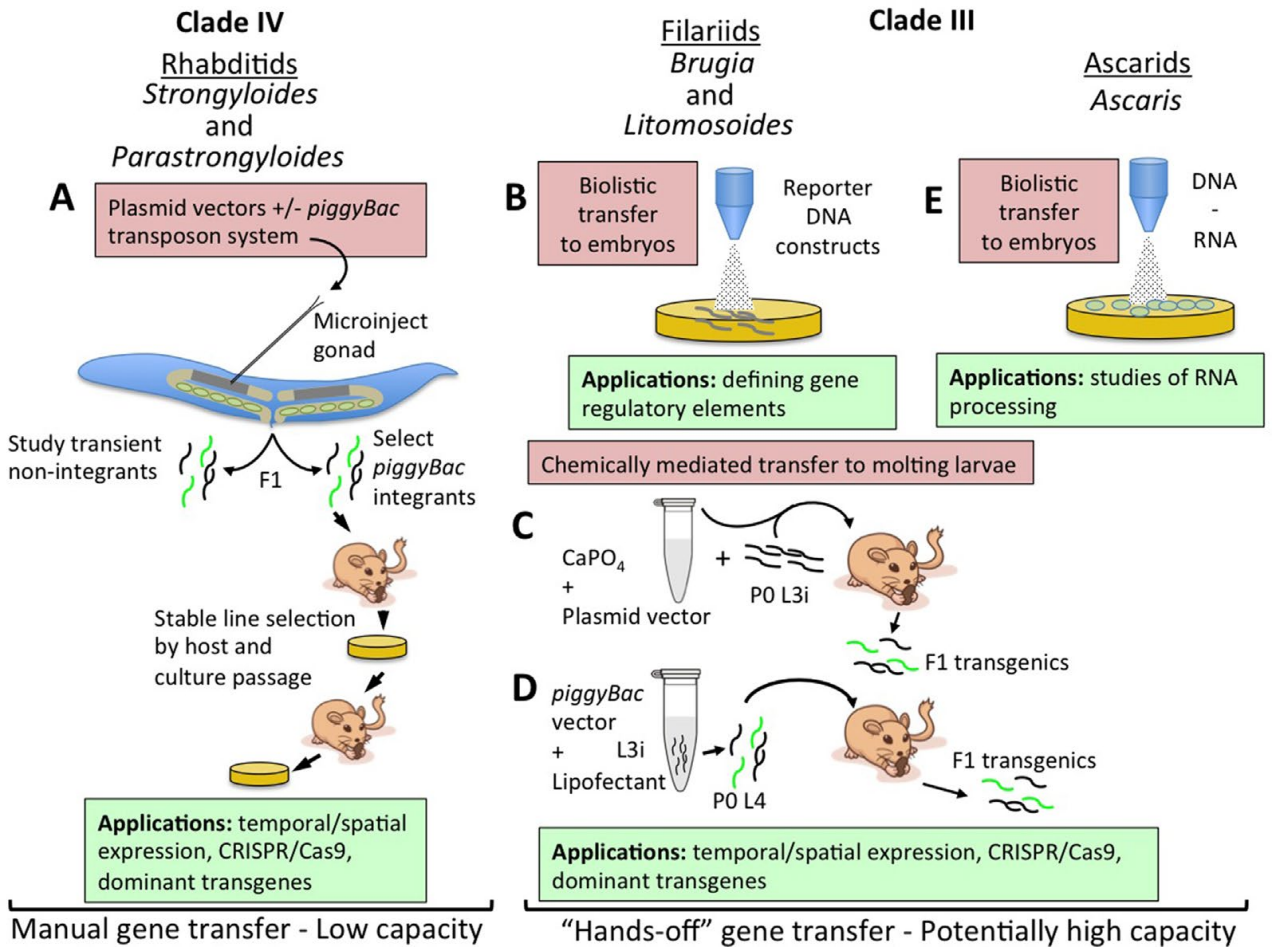
Strategies for transfer of nucleic acids into parasitic nematodes. **(A)** Microinjection of plasmid vector constructs into the syncytial gonads (shaded area of distal gonad arm in cartoon) of parental free-living female *Strongyloides* and *Parastrongyloides*, with or without transposon-mediated integration. Transiently transformed F1 larvae may be investigated for reporter expression patterns or phenotypes induced by dominant transgenes or CRISPR/Cas9-mediated gene disruption or editing. Lines of parasites with transgenes integrated by the co-injected *piggyBac* transposon system may be selected and maintained by alternating host and culture passage. **(B)** Biolistic transfer of reporter DNA constructs into *Brugia malayi* embryos within adult female worms has enabled definition of basic gene regulatory elements and has identified elements for ecdysteroid signaling in this parasite. Embryos transformed in this manner have thus far been incapable of further development. **(C)** Chemically mediated vector transfer in *B. malayi* by co-injection of parental infective third-stage larvae (L3i) into susceptible gerbils. Transgenic adult parasites and transgenic F1 microfilariae result from this procedure. **(D)** Refinement of chemically mediated transfection involving lipofectant transfer of plasmid vectors with the *piggyBac* transposon system to parental L3i of *B. malayi* undergoing the molt to L4 in culture and then inoculated intraperitoneally in to susceptible gerbils. F1 transgenic microfilariae include integrants and may prove amenable to selection of stable lines by mosquito and gerbil passage. **(E)** Biolistic transfer of DNA and RNA into embryos of *Ascaris suum*. This approach has enabled studies of RNA processing in these parasites.

Parasites in the genus *Strongyloides* and *Parastrongyloides* were the first to be successfully transformed with plasmid-based constructs (Lok and Massey, 2002; Grant et al., 2006a; Li et al., 2006; Li et al., 2011). The main factor allowing this advancement was these parasites’ unique ability to execute one or more generations of development as free-living males and females with their progeny in the soil (Viney and Lok, 2015; Grant et al., 2006b). These free-living cycles provide access to the adult germlines of *Strongyloides* and *Parastrongyloides* that is unavailable in obligately parasitic nematodes. Free-living generations of *Strongyloides* and *Parastrongyloides* can be maintained in agar plate culture using methods adapted from those used for the free-living nematode *C. elegans* (Grant et al., 2006a; Lok and Unnasch, 2013). *Parastrongyloides trichosuri* can execute an indefinite number of sequential free-living generations in plate culture, enhancing its utility as a subject for genetic study. Most crucially, the body plans of free-living female *Strongyloides* and *Parastrongyloides* and of *C. elegans* hermaphrodites are so similar that it was straightforward to adapt methods for gene delivery by **gonadal microinjection** from *C. elegans* (Fire, 1986; Fire et al., 1990a; Fire et al., 1990b; Mello et al., 1991; Mello and Fire, 1995) to the parasites (**Figure 1A**) (Lok and Massey, 2002; Grant et al., 2006a; Li et al., 2006; Li et al., 2011). A similar approach was used to achieve transgenesis in *Strongyloides stercoralis* by microinjection of plasmid constructs into the testes of free-living males (Shao et al., 2017). These approaches, involving transfer of plasmid-based vector constructs, allow promoter-regulated, tissue-specific transgene expression in F1 transformants (**Figure 1A**) (Grant et al., 2006a; Li et al., 2006; Junio et al., 2008; Li et al., 2011; Shao et al., 2017), and integration of transgene sequences into the chromosomes of *Strongyloides ratti* permits establishment of stable transgenic lines of this parasite (**Figure 1A**) (Shao et al., 2012). These methods have enabled studies of gene function in *Strongyloides* and *Parastrongyloides* that have revealed anatomical and temporal patterns of gene expression and essential functions of specific genes in development and survival of the pre-infective stages of these parasites (**Figure 1A**) (Massey et al., 2003; Grant et al., 2006a; Junio et al., 2008; Castelletto et al., 2009; Stoltzfus et al., 2012; Massey et al., 2013; Yuan et al., 2014a; Yuan et al., 2014b; Lei et al., 2017; Yuan et al., 2017).

Significant strides have also been made towards transgenesis in the filaria *Brugia malayi*. This parasite, and all filariae, are far more challenging subjects for molecular genetic study, as they have no free-living generation comparable to *Strongyloides* and related genera, and furthermore, unlike soil transmitted parasitic nematodes, their pre-infective stages require an arthropod vector for development. These barriers notwithstanding, transient DNA transformation of *B. malayi* (Higazi et al., 2002; Higazi and Unnasch, 2013) and *Litomosoides carinii* (Jackstadt et al., 1999) has been achieved by particle bombardment or biolistics (**Figure 1B**). In the case of *B. malayi*, the primary object of this approach has been to investigate the structure and function of gene regulatory sequences by transforming embryos within the uteri of female worms with plasmid constructs encoding fluorescent reporters linked to wild-type or mutant regulatory sequences, promoters mainly, and evaluating function by quantifying reporter activation. These bombarded embryos are not developmentally competent, but this approach has, nevertheless, yielded a wealth of basic information on the structural motifs required for promoter activity (Shu et al., 2003; Higazi et al., 2005; de Oliveira et al., 2008; Bailey et al., 2011), trans-splicing (Higazi and Unnasch, 2004; Liu et al., 2007; Liu et al., 2010a), and operon function (Liu et al., 2010b) in *B. malayi*. This approach to transient transgenesis has also revealed the function of microRNAs in regulation of *B. malayi* genes (Liu et al., 2015), the presence of tetracycline regulatable promoters (Liu et al., 2011), and the transcriptional targets of ecdysone signaling in this parasite (Tzertzinis et al., 2010; Liu et al., 2012). Notably, the approach of transiently transfecting embryos has also been applied to *Ascaris suum* (**Figure 1E**) (Davis et al., 1999).

Stable, heritable transformation has been achieved in *B. malayi* by chemically mediated transfer of plasmid vector constructs into developing infective third-stage larvae (L3i; **Figures 1C, D**). Initial applications of this approach involved forming calcium phosphate co-precipitates with plasmid DNA constructs encoding a luciferase (GLuc) reporter (Xu et al., 2011). Mosquito-derived infective third-stage larvae (iL3) were then co-injected along with the calcium phosphate plasmid DNA co-precipitate into the peritoneal cavities of susceptible gerbils. Substantial proportions of *B. malayi* adults and their microfilarial progeny recovered from gerbils inoculated with transduced iL3 expressed GLuc, proving inheritance of the reporter transgene in the F1 generation (**Figure 1C**). This method was subsequently refined such that DNA transfer was achieved by lipofection of mosquito-derived L3i of *B. malayi* that were undergoing the L3i-L4 molt in co-culture with bovine embryo skeletal muscle cells (Liu et al., 2018). Such cultured larvae co-transformed with GLuc and reporters encoding fluorescent proteins and flanked by inverted tandem repeats specific for the *piggyBac* transposon system as well as a plasmid encoding the *piggyBac* transpose were capable of establishing patent infection in susceptible gerbils (**Figure 1D**). Analysis adult *B. malayi* and F1 microfilariae revealed transgene expression and also integration of transgene sequences into the parasite genome. Altogether, this represents a remarkable advancement in functional genomic methodology for filariae and for all parasitic nematodes. For the former, it marks the first integrative transformation of *B. malayi*, the most widely used model in experimental filariasis, and the first deployment of fluorescent reporters that will allow rapid visual selection of transgenic parasites and assessment of temporal and anatomical patterns of transgene expression. For all parasitic nematodes, the work on *B. malayi* identifies chemically mediated DNA transfer as an alternative to gonadal microinjection as a means of transgenesis. This method has the advantage of DNA transfer into the larval germline, making it applicable to obligate parasitic nematodes in which the adult germline is not as easily accessible as it is in the free-living adults of *Strongyloides* and related parasites. **Chemically mediated gene transfer** has the additional and paramount advantage of being a “hands-off” method not requiring the meticulous and painstaking process of microinjection and having the potential for high capacity transformation of hundreds or thousands of parasites in a single replication of the procedure (**Figure 1B–E**). The common factor in success of the two chemically mediated approaches used to date in *B. malayi* (Xu et al., 2011; Liu et al., 2018) appears to have been that DNA transfer was undertaken during an intermolt period, in this case the L3–L4 molt, in which the nascent cuticle was transiently competent to take up either the calcium phosphate–DNA co-precipitate or the lipofectamine–DNA micelles. Parasitic nematodes such as hookworms or trichostrongyles and related rodent parasites such as *Nippostrongylus brasiliensis* and *Heligmosomoides polygyrus*, whose pre-parasitic larvae develop in the soil or on herbage, would seem to be particularly amenable to this approach, as they can be cultured through two molt cycles (L1–L3) in simple laboratory media. The same is true of *Strongyloides* and related genera, where the combination of life cycles containing both free-living and parasitic generations and a hands-off, high-capacity method of DNA transfer would make a particularly powerful model for molecular genetics in parasitic nematodes.

## Future Directions in Transgenesis for Parasitic Nematodes

### Gene Editing *via* Transgenesis in Pre-Parasitic Larvae of Soil-Transmitted Parasitic Nematodes

The microinjection-based methods of gene transfer into free-living females of *Strongyloides* spp. and of *Parastrongyloides trichosuri* have not been adapted to obligately parasitic nematodes whose pre-parasitic larvae develop in and are transmitted from the soil or vegetation and whose adult stages reside solely in vertebrate animal host. These include the hookworms and trichostrongyles of paramount importance to human medicine and animal agriculture. While the adult germlines of these parasites are not readily accessible for gene transfer, the rudimentary gonads of their pre-parasitic larvae, which are easily cultured in the laboratory, are accessible. Certainly, the chemically mediated and lipofectant-facilitated systems of gene transfer that have been so successful for *ex vivo* iL3 of *Brugia malayi* (Xu et al., 2011; Liu et al., 2018) should be investigated as possible approaches to gene transfer into hookworms and trichostrongyles of humans and domestic animals and their rodent counterparts that are important subjects for immunological research (Bouchery et al., 2017).

In addition to these “hands-off” approaches to gene transfer, it is also possible to transfer CRISPR/Cas9 elements into pre-parasitic larvae of soil-transmitted parasites by microinjection (Adams et al., 2019). Mutations were targeted to the *rol-6* ortholog in *S. stercoralis* by microinjecting a nucleoprotein comprising Cas9 and a target specific gRNA along with lipofectamine into the body cavities of iL3. This approach produced P0 mutant larvae exhibiting a classic “roller” phenotype identical to that seen in *rol-6* mutants of *C. elegans*. This approach might be adapted to the pre-infective larvae of other soil-transmitted parasitic nematodes as well.

### Deployment of “Hands-Off”, High-Capacity Gene Transfer in *Strongyloides* and Other Soil-Transmitted Parasitic Nematodes

A major shortcoming of gene transfer by gonadal microinjection in *Strongyloides* and related parasites has been the limited numbers of F1 transgenic larvae that can be produced by this approach (**Figure 1A**). This creates difficulties in amassing samples of transgenic larvae of sufficient size to achieve statistical power and is a particular hurdle to the establishment of transgenic lines of *S. stercoralis*, where the most susceptible rodent host, the gerbil, requires an approximate minimum of 100 transgenic larvae to mount a patent infection yielding transgenic F2 progeny. This situation calls for a concerted effort to achieve chemically or lipofectant-mediated gene transfer into pre-infective larvae of *Strongyloides* spp. in a manner similar to the successful methods now available for *ex vivo* iL3 of *Brugia* spp. (Xu et al., 2011; Liu et al., 2018). It seems likely that these “hands-off” methods (**Figure 1B–E**) could be similarly deployed for gene transfer into pre-parasitic larvae of hookworms, trichostrongyles, and their rodent-infecting counterparts.

## CRISPR/Cas9 Mutagenesis is Feasible in Parasitic Nematodes

DNA transformation of parasitic nematodes opens the formal possibility of deploying CRISPR/Cas9 for targeted mutagenesis as a means of directly assessing gene function in these pathogens. Proof of principle for **CRISPR/Cas9 mutagenesis** has been achieved in *Strongyloides stercoralis* and *Strongyloides ratti*. This includes preliminary evidence of precisely targeted double-stranded DNA breaks (DSB) and homology-directed repair with a co-transfected DNA template (Lok et al., 2017) and comprehensive demonstration and phenotypic evaluation of targeted disruption of *Ss-unc-22* and *Sr-unc-22* in *S. stercoralis* and *S. ratti*, respectively (Gang et al., 2017). Mutations have been created in *Strongyloides* by transducing free-living females with basic CRISPR elements, including gRNA, Cas9 codon-optimized for expression in *Strongyloides*, and selectable markers encoded in plasmid vectors (Gang et al., 2017; Lok et al., 2017) or by microinjecting gonads of free-living females with pre-formed nucleoproteins comprising recombinant Cas9 and *in vitro* transcribed gRNAs (Gang et al., 2017; Adams et al., 2019). To date, transduction of parental worms with plasmid-encoded CRISPR/Cas9 elements has resulted in more efficient mutagenesis than is seen with delivery of pre-formed nucleoproteins (Gang et al., 2017).

Informative phenotypes have been associated with both large deletions at those sites mediated by non-homologous end joining (NHEJ) and with insertional mutagenesis *via* homology-directed repair (HDR) of CRISPR/Cas9-induced DSB. Most notably, a prominent “twitcher” phenotype, which is exacerbated by nicotine exposure, results from CRISPR/Cas9 disruption of *Ss-unc-22* and *Sr-unc-22*. These genes, like their *C. elegans* ortholog, encode the intracellular muscle protein twitchin (Moerman and Baillie, 1979; Moerman et al., 1988; Gang et al., 2017). The *unc-22* phenotypes in *Strongyloides* spp. are highly reminiscent of the twitcher phenotype resulting from mutations in C. *elegans unc-22*. Notably, the *C. elegans* twitcher phenotype is dominant, occurring in both homozygous and heterozygous *unc-22* mutants. Homology-directed repair has been exploited to integrate short disrupting oligonucleotides containing multiple stop codons and novel priming sites for genotyping at the site of CRISPR/Cas9-induced DSBs (Lok et al., 2017) as well as a full reporter cassette encoding a fluorescent reporter that serves to both disrupt the target gene and to provide a convenient marker for mutant selection (Gang et al., 2017). In the latter instance, F1 larvae expressing the red fluorescent protein mRFPmars under the body wall-specific promoter for *Ss-act-2* from the repair template in an episomal array or from the template integrated into a CRISPR/Cas9 cut site in *Ss-unc-22* could be distinguished by PCR using primers specific for the repair template and the genomic region flanking the cut site and by the consistent pattern of body wall expression in integrants (Gang et al., 2017). Integrants in this case exhibited a uniform twitcher phenotype, providing proof of principle for the use of reporter-encoding repair templates to disrupt target genes by CRISPR/Cas9- and HDR-mediated integration.

Most significantly, CRISPR/Cas9-induced mutations in *S. stercoralis* are heritable and may be propagated by passage in gerbils, which constitute the most authentic rodent model of the entire spectrum of *S. stercoralis* infection and disease in humans (Nolan et al., 1993; Gang et al., 2017).

In addition to the studies of *Ss-unc-22*, which demonstrated that its encoded muscle component is required for normal motility of iL3 (Gang et al., 2017), CRISPR/Cas9 and the *Strongyloides* model have found application in studies of genes involved in the sensory biology of these infective larvae. Soil transmitted parasitic nematodes such as hookworms and *Strongyloides* spp. invade the host by skin penetration and develop as free-living pre-parasitic larvae in contaminated soil. iL3 of these parasites orient toward potential hosts using a complex of chemical and physical cues (Bryant and Hallem, 2018b; Castelletto et al., 2014). Orienting towards a thermal source is a behavior that many species of environmentally motile parasitic nematode iL3 use to acquire a host (Castelletto et al., 2014; Bryant and Hallem, 2018a; Bryant and Hallem, 2018b; Bryant et al., 2018). In *C. elegans*, thermal signals are received by receptor guanylate cyclases in sensory neurons of the amphidial complex, which are in contact with the external environment. These receptors signal through a heteromeric cGMP-activated cation channel, comprising TAX-2 and TAX-4 subunit proteins, that regulates polarization of the sensory neuron and potentiation of thermotactic responses (Komatsu et al., 1996; Bargmann and Mori, 1997; Komatsu et al., 1999). The *tax-4* ortholog in *S. stercoralis* has been disrupted by CRISPR/Cas9, and knockout iL3 exhibit diminished themotaxis towards 34°C compared to controls derived from parental worms receiving all CRISPR/Cas9 elements except the Cas9 endonuclease. This provides direct evidence that *tax-4* is required for normal thermotaxis by iL3 of this parasite (Bryant and Hallem, 2018a; Bryant and Hallem, 2018b; Bryant et al., 2018). Given the involvement of the TAX-2/TAX-4 channel in other sensory signaling pathways in free-living nematodes (Komatsu et al., 1996; Komatsu et al., 1999), it is likely that *S. stercoralis tax-4* knockouts will exhibit decrements in chemotactic as well as thermotactic behaviors when these mutants are subjected to the appropriate assays (Bryant and Hallem, 2018b; Bryant et al., 2018).

## Future Directions for CRISPR/Cas9 in Parasitic Nematodes

### Deployment of CRISPR/Cas9 in Filariae

Robust systems for both integrative (Liu et al., 2018) and non-integrative (Xu et al., 2011) transgenesis are now available for *Brugia malayi*. With these systems in place, targeted mutagenesis and gene editing in this important model of human filariasis are imminently possible. The toolkit for integrative transgenesis assembled by Liu et al. (Liu et al., 2018) comprises a set of multifunctional vectors encoding multiple elements of the *piggyBac* transposon system that could be readily adapted to encode CRISPR/Cas9 elements such as the Cas9 endonuclease, gene-specific gRNAs, and templates for homology-directed repair in a compact and efficient gene delivery system. The time is right for this advancement.

### Single-Copy Transgene Integrations

Among the very significant findings that arose from proof of principle for CRISPR/Cas9 in *Strongyloides* (Gang et al., 2017) was that full gene coding sequences, in this case an *Ss-act-2* transcriptional reporter encoding mRFPmars, can be integrated into the parasite genome at CRISPR/Cas9 cut sites by homology-directed repair. This approach provides a distinct advantage over current transposon-based methods for transgene integration, which integrate multiple transgene copies at random sites in the genome. Integrations by *piggyBac* typically favor coding sequences, creating the possibility of confounding insertional mutagenesis (Shao et al., 2012; Lok, 2013).

### Regulatable CRISPR/Cas9

The capability to target transgene integrations to precise locations within the *Strongyloides* genome by CRISPR/Cas9 creates the potential to establish lines of these parasites that stably express the Cas9 endonuclease from single transgene copies. Parasites from such lines could subsequently be transformed to express gene-specific gRNAs to disrupt target genes and assess resulting phenotypes. Constitutive expression of Cas9 in *C. elegans* exerts some deleterious effects on fitness of the transgenic worms (Waaijers et al., 2013). Likewise, disrupting genes essential for invasion or establishment of the worms in mammalian hosts would prevent propagation of mutants and assessment of phenotypes affecting host infectivity, or crucial host–parasite interactions. A solution to these problems could be conditional or inducible Cas9 expression from integrated transgene sequences in stable parasite lines. We envision two possible approaches to regulating Cas9 expression in this context. The first would be to express Cas9 under promoters that are activated in the presence of a small molecule regulator such as tetracycline (Tet). Tet-responsive genes have been identified in *Brugia malayi* (Liu et al., 2011; Wurmthaler et al., 2019), and a tet-regulated promoter has been deployed for inducible transgene expression in *C. elegans* (Liu et al., 2011; Wurmthaler et al., 2019). These findings argue for identifying Tet-responsive genes in *Strongyloides* that could be similarly incorporated into **regulatable transgene expression** systems generally and particularly for the regulated expression of Cas9 in stable parasite lines.

Another approach to regulatable expression of Cas9 and other transgene-encoded factors would be to fuse these to degradation domains that are stabilized in the presence of small molecules. Under these conditions, the transgene product would be stabilized in the presence of the small molecule but targeted to the proteasome for degradation in its absence. Such a system, incorporating the degradation domain of dihydrofolate reductase, which is stabilized in the presence of the folate-targeting drug trimethoprim, has been used to regulate transgene expression in *Plasmodium falciparum* (Muralidharan et al., 2011), and it has shown promise in preliminary experiments with transgenic Strongyloides (Lok et al., 2017).

## Transgenes With Dominant Mutations are Tools for Functional Genomic Study in Parasitic Nematodes.

### Functions of Genes Encoding a Variety of Catalytic or Regulatory Proteins may be Interrogated With Dominant Interfering Transgenes

**Figure 2:**
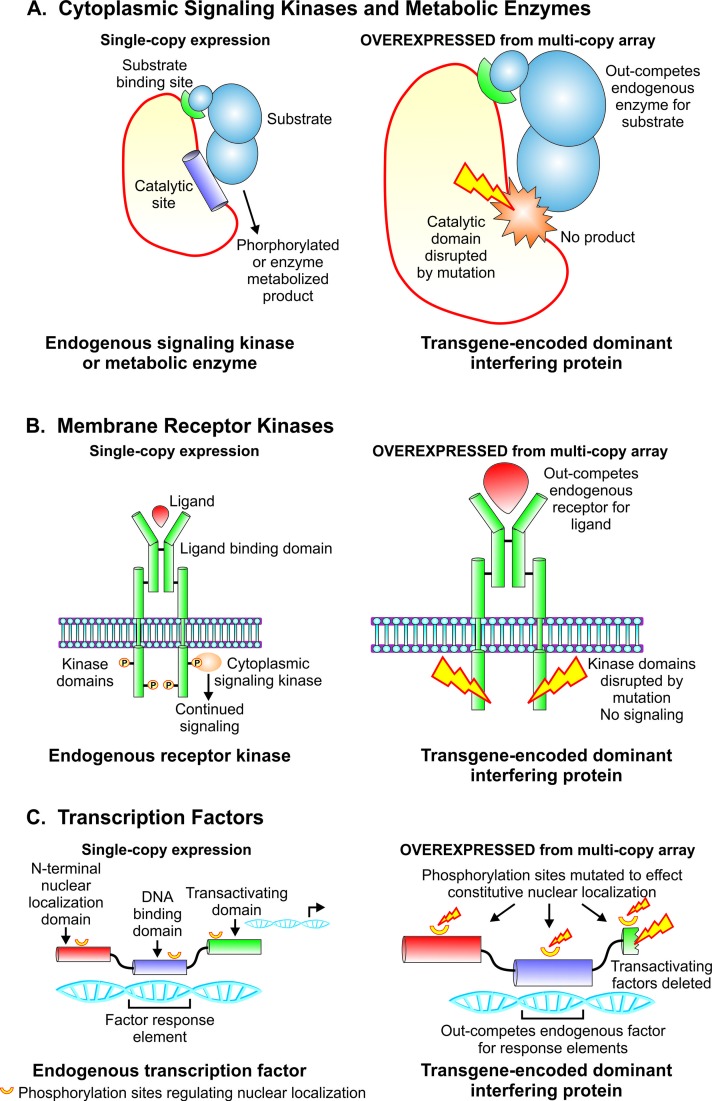
Strategies for creating dominant interfering transgene constructs to study functions of catalytic, signaling, and gene regulatory proteins. **(A)**. Strategy for dominant interfering transgenes targeting functions of metabolic enzymes and cytoplasmic signaling kinases. Overexpression of mutant construct with intact substrate binding domain and catalytic site disrupted by mutation serves to outcompete endogenous gene product for substrate while yielding no phosphorylated or enzyme metabolized product. **(B)**. Design of dominant interfering transgenes targeting functions of membrane receptor kinases. Ablation of the kinase domain by mutation, an intact ligand binding domain and overexpression combine to out-compete the endogenous receptor kinase for ligand while failing to phosphorylate downstream cytoplasmic signaling elements. **(C)**. Design of dominant interfering transgenes targeting the action of transcription factors. Overexpression of a mutant transgene encoding an intact DNA binding domain, mutations in phosphorylation sites to effect constitutive nuclear localization, and disruption of the transactivating domain serve to outcompete the endogenous transcription factor for genomic response elements while failing to execute gene regulatory function.

Prior to the advent of targeted mutagenesis, transgenesis in *Strongyloides* spp. enabled a functional genomic approach based on expression of transgenes encoding mutant proteins designed to exert a dominant interfering effect on the target of interest. This approach may be used to suppress the functions of catalytic, signaling, and regulatory proteins that comprise separate functional domains and domains that bind to substrates, ligands, or DNA (**Figure 2**). The strategy holds that when overexpressed from multi-copy arrays relative to the single-copy endogenous target in the subject organism, transgene-encoded mutant proteins that have disrupted functional domains and intact binding domains will out-compete their endogenous wild-type counterparts for binding partners but fail to undertake their putative functions, thus resulting in dominant loss of function in the target gene of interest. Basic mechanisms for this approach to simulate loss of function in target genes are illustrated for cytoplasmic signaling kinases and metabolic enzymes (**Figure 2A**), membrane receptor kinases (**Figure 2B**), and transcription factors (**Figure 2C**).

### A Dominant Transgene Approach has been Used to Investigate the Function of *Ss*-DAF-16 in *S. Stercoralis*

The gene encoding this transcription factor is the ortholog of *daf-16* in *C. elegans*, which encodes a forkhead transcription factor that is regulated by insulin-like signaling and controls lifespan and the switch between dauer and continuous larval development (Lin et al., 1997; Ogg et al., 1997; Lee et al., 2001; Massey et al., 2003; Massey et al., 2006). A loss-of-function approach to interrogating the function of *Ss-daf-16* employed a dominant interfering construct. In this construct, AKT phosphorylation sites necessary for cytoplasmic localization of the protein were disrupted and the C-terminal domain of the molecule was truncated sufficiently to delete predicted transactivator binding sites (**Figure 2C**). Sequence encoding the DNA-binding domain of the mutant *Ss*-DAF-16 was left intact. The putative **dominant interfering transgene** also fused the coding sequence of the mutant *Ss-daf-16* with that of *gfp* to confirm constitutive nuclear localization of the encoded protein (Castelletto et al., 2009). This construct, therefore, was designed to express a mutant form of *Ss*-DAF-16 that is constitutively localized to the nucleus, but lacks the transactivating functions of the wild-type transcription factor. We also assume that the mutant transgene, like other plasmid-encoded transgenes introduced to nematodes by gonadal microinjection, will be incorporated into a multi-copy episomal array (Mello et al., 1991) and therefore overexpressed relative to the endogenous single-copy target gene (**Figure 2C**). Indeed, a proportion of F1 progeny of free-living female *S. stercoralis* microinjected with the mutant *Ss-daf-16* construct exhibited pronounced nuclear localization of GFP and a range of phenotypes including severe defects in intestinal morphology, loss of secretory granules from intestinal cells, retention of rhabditiform pharyngeal morphology in third-stage larvae (L3), and initiation of an aberrant L3–L4 molt (Castelletto et al., 2009). These last two phenotypes are particularly noteworthy given that 100% of wild-type progeny of free-living *S. stercoralis* females arrest their development as infective third-stage larvae, which have filariform, as opposed to rhabditiform morphology (Schad, 1989; Viney and Lok, 2015). In contrast to parasites expressing the putative dominant interfering construct, F1 progeny transformed with a control construct fusing the wild-type *Ss-daf-16* coding sequence to *gfp* exhibited normal intestinal architecture, with intestinal cells replete with secretory granules, filariform pharyngeal morphology in L3, and no evidence of L3–L4 molting (Castelletto et al., 2009).

### A Dominant Interfering Transgene Construct was Used to Assess the Function of *Ss-riok-1* in *S. Stercoralis*

*Ss-riok-1* encodes a RIO protein kinase homologous to RIOK-1 in *C. elegans* (Yuan et al., 2014b). RIO kinases are conserved in eukaryotic organisms and are essential for ribosomal biogenesis and cell cycle progression (Angermayr et al., 2002; Widmann et al., 2012; Yuan et al., 2014b). The putative dominant interfering construct encoded *Ss-riok-1* with a D282A mutation in its catalytic site that served to disrupt kinase activity. Sequence encoding the substrate binding site of *Ss-riok-1* was left intact in the putative dominant interfering construct (**Figure 2A**). As with the *Ss-daf-16* constructs described above, both the wild-type and mutant constructs encoding *Ss*-RIOK-1 fused its coding sequence to that of *gfp* to confirm an authentic pattern of expression under the *Ss-riok-1* promoter. As expected, both mutant and wild-type constructs were expressed in head and tail neurons and hypodermal cells of transgenic *S. stercoralis* larvae (Yuan et al., 2014b; Yuan et al., 2017). It was also expected that, by virtue of its situation in a multi-copy episomal array, the mutant protein expressed from the dominant transgene construct would outcompete endogenous single-copy Ss-RIOK-1 for substrate binding but fail to exert its kinase function (**Figure 2A**). Larvae transformed with the construct encoding the kinase dead *Ss*-RIOK-1 exhibited profound decrements in motility and a significant blockade in development to the infective third-stage. By contrast, *S. stercoralis* larvae expressing a control construct encoding a fusion of *Ss*-RIOK-1 and GFP exhibited typical progressive motility and developed normally to the iL3 (Yuan et al., 2017). The study of *Ss-riok-1* function included an additional and crucial control experiment in which co-expression of wild-type *Ss*-RIOK-1 with the kinase-dead mutant served to rescue the motility and developmental phenotypes to a degree roughly proportional to concentration of wild-type vector plasmid microinjected into parental free-living female worms (Yuan et al., 2017). This important control experiment served to confirm the specificity of the dominant interfering transgene’s effects on *Ss*-RIOK-1 function.

## Future Directions in the Use of Dominant Transgene Constructs to Study Gene Function in Parasitic Nematodes

### Controlling Experiments Involving Dominant Interfering Constructs

Both studies employing dominant interfering constructs to evaluate specific gene function in *S. stercoralis* (Castelletto et al., 2009; Yuan et al., 2017) controlled for confounding effects of transgene overexpression by comparing phenotypes in cohorts of parasites overexpressing fusions of wild-type target proteins fused to GFP to those exhibited by parasites expressing mutant ones. This is crucial given the possibility that overexpression alone can, in some instances, produce dominant effects in transgenic organisms (Chandler and Werr, 2003). Another crucial control introduced in the study of *Ss*-RIOK-1 function was rescue of dominant interfering phenotypes by co-overexpression of the wild-type protein. In this case, the dose dependency of rescue was established by assessing frequencies of mutant phenotypes in F1 larvae derived from parental females microinjected with the mutant construct along with increasing concentrations of vector plasmid encoding the wild-type protein fused to GFP. There was a decreasing trend in the frequency of phenotypes with increasing concentration of wild-type vector plasmid, confirming the specificity of the mutant constructs dominant effect (Castelletto et al., 2009; Yuan et al., 2017). Inclusion of both of these essential controls will be crucial to the correct interpretation of data in future experiments with dominant interfering transgene constructs.

### Deployment of Dominant Transgenes Incorporating Heterologous Activator or Suppressor Domains

To date, dominant interfering effects have been achieved with transgenes encoding the target gene with synthetic loss-of-function mutations in functional domains and wild-type sequence in DNA, protein, or substrate binding sites to facilitate competition for binding partners with the product of the endogenous target gene (**Figure 2**). Another approach to creating dominant interfering constructs targeting transcription factors in parasitic nematodes is to design the constructs to encode a chimeric protein comprising a heterologous transcriptional repressor domain fused to the DNA binding domain of the target factor. This serves to direct a strong repressor to promoters of genes regulated by the target transcription factor. An example of this approach is the use of the *Drosophila* ENGRAILED homeodomain to impart repressor function to the transcription factors, thereby creating loss-of-function phenotypes in both invertebrate and mammalian models (Badiani et al., 1994; John et al., 1995; Tolkunova et al., 1998; Chandler and Werr, 2003). Conversely, transgene constructs comprising viral activator domains such as VP16 from herpes simplex virus can activate target genes when fused to GAL-4 (Sevin-Pujol et al., 2017). The specific gene targeting capabilities of nucleoproteins comprising an inactive form of the Cas9 endonuclease, dCas9, and gRNAs can also be harnessed to tether repressor domains such as KRAB or activators such as VP64 to precise genome loci to achieve transcriptional repression (CRISPRi) or activation (CRISPRa) of target genes (Dominguez et al., 2016).

## Conclusions

Transgenesis in *Strongyloides* and related parasitic nematodes in phylogenetic Clade IV and in the filaria *Brugia malayi* within Clade III opens possibilities for unprecedented studies of gene function in these pathogens that degrade the health of hundreds of millions of people. These approaches have enabled experiments employing dominant interfering transgenes to reveal functions of genes essential to ribosomal biogenesis and to morphogenesis and developmental arrest of infective larvae in *Strongyloides stercoralis*. Moreover, transgenesis has recently supported proof of principle for targeted mutagenesis *via* CRISPR/Cas9 in *Strongyloides* spp. Recent successes with integrative transgenesis in *Brugia malayi* create the possibility that similar studies of gene function will be possible in the filariae. Recent work with *Brugia* (Liu et al., 2018) and *Strongyloides* (Adams et al., 2019) illustrate the potential of lipofectant-mediated gene transfer, alone or in combination with microinjection, to facilitate applications of transgenesis and CRISPR/Cas9 gene disruption and editing to functional genomic study of a wide range of obligately parasitic nematodes with vector borne and free-living pre-parasitic larvae.

## Key Concepts

**Gonadal microinjection** refers to a method of gene transfer whereby solutions of plasmid or linear transgene constructs are injected into the germlines of parental male or female worms. Nematode gonads frequently have syncytial regions that comprise germ cell nuclei within a common cytoplasm. Infusion of transgene construct solutions into these gonadal syncytia results in significant numbers of transgenic oocytes or sperm.

**Chemically mediated gene transfer**, in the present context, involves gene transfer across the cuticles or exposed cellular surfaces of nematodes facilitated by incorporating transgene DNA into calcium phosphate co-precipitates or lipofectant micelles. Presenting these DNA preparations during intermolt periods of developing larvae, presumably when nascent cuticles are competent to take them up, may be crucial to this process.

**Regulatable transgene expression** is a strategy that allows transgene expression to be activated by the experimenter. This may be accomplished by using promoters that respond by activation to small molecules such as tetracycline or by coupling transgene encoded proteins of interest to degradation domains that are stabilized in the presence of some small molecule and degraded in the proteasome in its absence.

**CRISPR/Cas9.** Based on a prokaryotic defense response to foreign DNA, CRISPR/Cas9 harnesses sequence-specific targeting of the Cas9 endonuclease to precise gene loci by short “guide RNAs” to induce double-stranded DNA breaks. These may be repaired by non-homologous end joining, leaving random insertions or deletions, or by homology-directed repair, which can serve to incorporate a synthetic DNA repair template.

**Dominant interfering transgenes** encode mutations that ablate functional domains of a recombinant protein of interest, but leave crucial protein- or DNA-binding domains intact. When overexpressed, products of these dominant interfering transgenes outcompete the endogenous wild-type gene product for binding partners but fail to execute their designated functions, creating a loss-of-function phenotype in the subject.

## Author Contributions

JL formulated the concepts in the review, searched and analyzed the articles referenced, wrote all drafts of the text and designed and drafted the figure, which was then rendered by a graphic artist in the author’s department and who is acknowledged in the paper.

## Funding

The author has received support from grants AI50688, AI105856, AI144572, and OD P40-10939 from the US National Institutes of Health and from a grant from the University of Pennsylvania Research Foundation.

## Conflict of Interest Statement

The authors declare that the research was conducted in the absence of any commercial or financial relationships that could be construed as a potential conflict of interest.
